# Bioinformatics analysis of JUP in patients with acute myocardial infarction and its potential application in clinical prognostic evaluation

**DOI:** 10.3389/fcvm.2025.1531309

**Published:** 2025-05-13

**Authors:** Xinyue Deng, Ailing Shen, Leiying Jiang

**Affiliations:** Department of Clinical Laboratory, Suzhou Hospital of Integrated Traditional Chinese and Western Medicine, Suzhou, China

**Keywords:** JUP, AMI, adverse events, prognosis, PG

## Abstract

**Background:**

Junctional Plakoglobin (JUP) is a critical protein involved in intercellular junctions, playing a significant role in maintaining the structure and function of myocardial cells. However, the expression of JUP in acute myocardial infarction (AMI) and its potential applications in prognostic evaluation of patients remain underexplored. This study aims to investigate the expression levels of JUP in AMI patients and its association with clinical prognosis through bioinformatics analysis.

**Methods:**

A total of 164 patients with acute myocardial infarction admitted from January 2022 to January 2024 were selected as the study subjects. They were divided into an MACE group and a non-MACE group based on the occurrence of adverse prognostic events. Clinical data and myocardial tissue samples from patients post-percutaneous coronary intervention (PCI) were collected. The expression levels of JUP in myocardial tissue were assessed using quantitative real-time PCR (qPCR), and the functional role of the JUP gene in the prognosis of acute myocardial infarction was analyzed. The impact of JUP expression levels on the prognosis of AMI patients was evaluated using Kaplan-Meier method and Cox Proportional Hazards Model.

**Results:**

The expression level of JUP in the MACE group was significantly lower than that in the Non-MACE group (*P* < 0.05). The results of the Cox Proportional Hazards Model further indicated that TnI levels (HR = 12.512, 95% CI: 1.622–96.507, *P* < 0.05), multi-vessel disease (HR = 0.300, 95% CI: 0.108–0.834, *P* < 0.05), and myocardial JUP levels (HR = 0.234, 95% CI: 0.065–0.846, *P* < 0.05) were independent predictive factors for post-PCI outcomes in patients with acute myocardial infarction. Kaplan-Meier method revealed a significant association between low JUP expression and adverse prognosis in AMI patients (*P* < 0.05). ROC curve showed that multi-vessel disease (AUC = 0.6548, Sensitivity = 64.29%, Specificity = 66.67%), TnI (AUC = 0.8316, Sensitivity = 40.71%, Specificity = 91.67%), and myocardial JUP (AUC = 0.8299, Sensitivity = 75.00%, Specificity = 84.29%) could all predict the risk of major adverse cardiac events (MACE) after PCI in AMI patients.

**Conclusion:**

The expression level of JUP is decreased in patients with acute myocardial infarction and is closely associated with adverse prognostic outcomes. JUP may serve as a potential biomarker for assessing prognosis in AMI patients, providing new insights for the development of personalized treatment strategies.

## 1 Introduction

Acute myocardial infarction (AMI) is a common and severe pathological condition within cardiovascular diseases, resulting from the acute interruption of coronary blood supply, leading to ischemic necrosis of the myocardium, with a very high mortality and morbidity rate (1). Although the application of emergency percutaneous coronary intervention (PCI) has significantly improved early treatment outcomes for AMI patients, there remains a high incidence of major adverse cardiovascular events (MACE) post-surgery, including heart failure, reinfarction, and sudden cardiac death. Consequently, effectively predicting and preventing adverse postoperative outcomes has become one of the focal points of clinical research. Identifying reliable biomarkers for predicting adverse events following AMI is of considerable clinical significance.

Currently, several biomarkers are utilized for the diagnosis and prognostic evaluation of acute myocardial infarction (AMI), including cardiac troponin I (TnI), creatine kinase (CK), and N-terminal pro-b-type natriuretic peptide (NT-proBNP). These biomarkers have demonstrated significant utility in assessing myocardial injury and cardiac function. However, their predictive sensitivity and specificity have certain limitations, particularly in evaluating long-term postoperative prognosis. Advances in bioinformatic analysis techniques provide new tools for the screening and validation of disease-related genes. This study utilizes datasets from the GEO database, combined with multi-omics approaches, to identify genes with significant differences between AMI patients and healthy controls and further explore the potential biological functions of these genes and their application value in post-operative prognosis.

In this study, we specifically focus on junctional plakoglobin (JUP). Also known as γ-catenin (2), JUP is a crucial component of adherens junctions and is widely present in the intercalated discs of myocardial cells (3). JUP plays a vital role in the heart by maintaining the structural integrity of cardiomyocytes and ensuring signal transduction and mechanical coupling between them (4). Recent studies have indicated that mutations or abnormal expression of the JUP gene are closely associated with various diseases, including prostate cancer (5), ovarian cancer (6), oral squamous cell carcinoma (7), gastric cancer (8, 9), arrhythmogenic right ventricular cardiomyopathy (ARVC) (10, 11), Naxos disease (12), and hypertrophic cardiomyopathy (13). This highlights its relevance in cancer and underscores its importance in maintaining normal cardiac function. However, the specific role of JUP in acute myocardial infarction (AMI) and its potential impact on patient prognosis remain insufficiently explored.

## 2 Materials and methods

### 2.1 Research subjects

**Figure 1:**
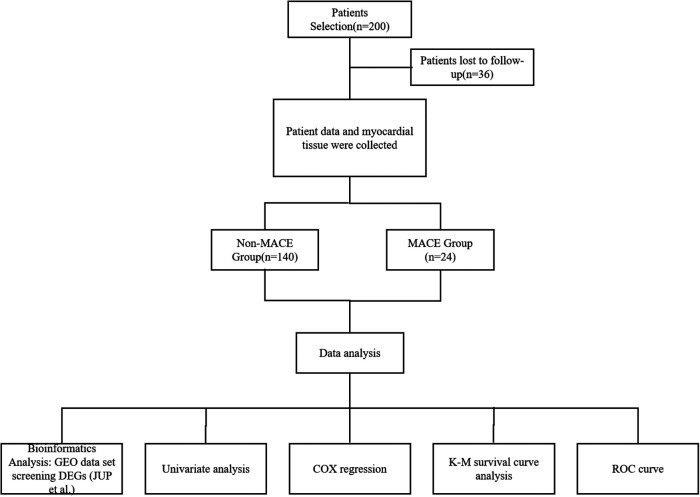
The flowchart of the study.

A total of 200 patients with acute myocardial infarction admitted from January 2022 to January 2024 were selected as the study objects. After one year's follow-up, a total of 36 patients with missing data were not included in this study, and 164 patients were divided into event group and non-event group according to the occurrence of adverse prognostic events. Myocardial tissue samples and clinical data were collected. This study was approved by the Medical Ethics Committee of our hospital (Approval No. 2024-021) and adhered to the principles outlined in the Declaration of Helsinki. Informed consent was obtained from all patients or their relatives throughout the experimental process. As shown in Figure 1.

Inclusion criteria: (1) Meeting the criteria outlined in the Fourth Universal Definition of Myocardial Infarction (2018) (14).

Exclusion criteria: (1) Presence of old myocardial infarction or heart failure due to other causes; (2) Coexisting severe cerebrovascular disease or liver and kidney dysfunction; (3) Malignancies or hematological diseases; (4) Severe gastrointestinal ulcers with bleeding; (5) Coagulation disorders. All patients were first-time cases, preoperatively treated with loading doses of aspirin, ticagrelor, and atorvastatin, and underwent PCI intervention within 12 h. Postoperatively, they received basic treatments including antithrombotic therapy, anticoagulation, vasodilation, and plaque stabilization.

### 2.2 Methods

Bioinformatics tools were utilized to analyze and predict differentially expressed genes (DEGs) associated with acute myocardial infarction, and quantitative real-time PCR (qPCR) was employed to assess the expression levels of JUP in myocardial tissue. All patients or their relatives provide written informed consent prior to PCI, consenting to the use of any cardiac muscle tissue obtained during the procedure for pathological diagnosis and subsequent research purposes. Myocardial tissue samples are collected during emergency PCI as part of routine diagnostic procedures. The primary purpose of tissue acquisition is rapid intraoperative pathological assessment to rule out other myocardial pathologies or to confirm the extent of infarction. After completing the necessary clinical diagnostic analysis, any remaining tissue was anonymized and used for this study. Immediately after collection, the specimen was divided into two parts: one part was used for routine pathological examination, processed into paraffin embedded sections, and archived; The other part is stored in liquid nitrogen or −80 °C for RNA extraction and qPCR analysis. Clinical data were collected from all enrolled patients upon admission, including age, gender, BMI, door-to-balloon time, hypertension, heart rate, atrial fibrillation, smoking history, diabetes, left ventricular ejection fraction (EF%), LDL-C, CK, TnI, and postoperative TIMI grading, as well as the number of affected vessels. Major adverse cardiovascular events (MACE) were considered important follow-up indicators for PCI prognosis, encompassing cardiogenic death, myocardial infarction, angina pectoris, heart failure, repeat revascularization, malignant arrhythmias, and stent thrombosis. Door-to-balloon time refers to the duration from patient arrival at the interventional hospital to balloon dilation during the procedure.

#### 2.2.1 Sample size calculation

G*POWER 3.9 was used to calculate the sample size. The expression level of JUP in the two groups was used as the basis for predicting the sample size. Based on the absence of relevant studies, the comparison between the two groups with or without MACE was conducted. Therefore, the sample size was calculated according to the following assumed values. Effect size d = 0.8, α error prob = 0.05, Power(1-β err prob) = 0.80, Allocation ratio N2/N1 = 0.173 [The study showed that the incidence of MACE was 17.3% (15)]. A total of 100 patients were calculated, including 15 patients in the MACE group and 85 patients in the non-MACE group. A total of 164 patients were included in this study, meeting the sample size required for the study.

#### 2.2.2 Identification of differentially expressed genes (DEGs) in acute myocardial infarction

Acute myocardial infarction-related datasets GSE60993, GSE127853, GSE218474, and GSE249812 were obtained from the GEO database (https://www.ncbi.nlm.nih.gov/gds/). The Jvenn system (https://jvenn.toulouse.inrae.fr/app/example.html) was employed to generate a Venn diagram illustrating the intersection of differentially expressed genes (DEGs) between acute myocardial infarction patients and healthy controls across these datasets. Ultimately, five DEGs associated with acute myocardial infarction were identified: JUP, DUSP5, GLRX, VDAC3, and INSIG1, *P* < 0.05, |Log_2_FC| ≥ 1.

#### 2.2.3 Detection of JUP

Total RNA was extracted using an RNA extraction kit and converted to cDNA using a cDNA reverse transcription kit (Takara, China). qPCR was performed on ABI 7500 system (Applied Biosystems, California, USA) with 20 μl SYBR-Green PCR reaction system. It includes 1× SYBR-Green PCR main mixture (Takara), 10 ng cDNA and 100 nM specific primers for JUP and GAPDH, and the primers sequence is shown in Table 1. The reaction conditions were: pre-denaturation at 95 °C for 10 min, followed by 40 cycles, including denaturation at 95 °C for 15 s, annealing at 60 °C for 1 min, and extension at 72 °C for 30 s. Dissociation curves were drawn to confirm the singleness and specificity of the amplified products. The cyclic threshold (Ct) was analyzed using SDS 2.0 software (Applied Biosystems), and the relative expression level of JUP was quantified by comparing Ct with GAPDH as the internal reference. All experiments were repeated three times.

**Table 1:** Primer sequence of JUP.

Gene	Forward primer(5′ → 3′)	Reverse primer(5′ → 3′)
JUP	-CATACTCAGGTGCGGGCTAT-	-GGTGTATGTCTGCTGCCACT-
GAPDH	-AATGGGCAGCCGTTAGGAAA-	-GCGCCCAATACGACCAAATC-

### 2.3 Statistical analysis

Statistical analysis was performed using SPSS 20.0 (IBM Corp., Armonk, NY, USA). Normally distributed continuous data were expressed as mean ± standard deviation, and comparisons between two samples were conducted using the *t*-test. Categorical data were presented as *n*/%. The Kaplan-Meier method and Cox Proportional Hazards Model were employed to evaluate the risk factors for major adverse cardiovascular events (MACE) following PCI in AMI patients, with *P* < 0.05 considered statistically significant.

## 3 Results

### 3.1 Bioinformatics analysis of differentially expressed genes associated with acute myocardial infarction

**Figure 2:**
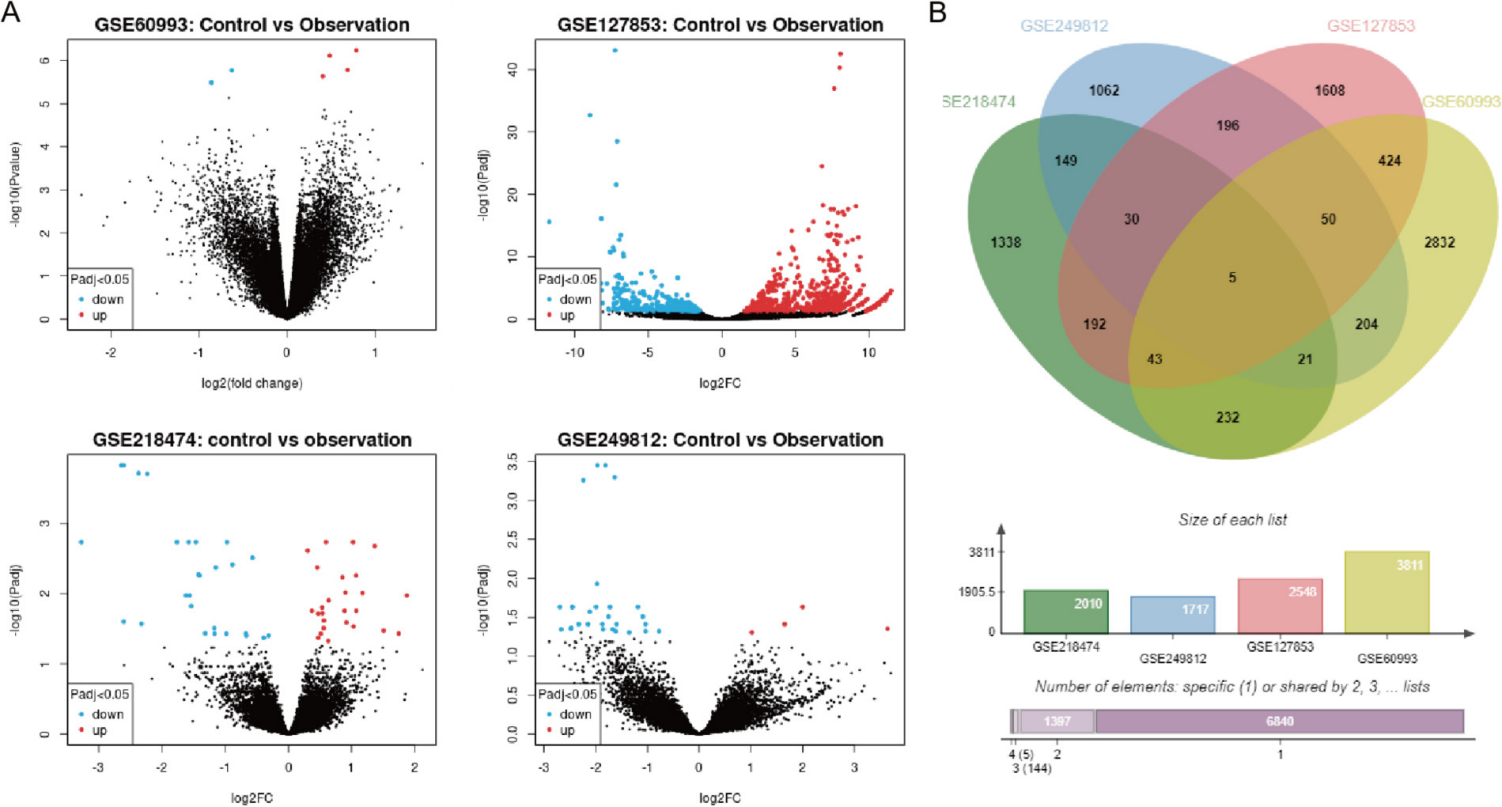
Bioinformatics results related to acute myocardial infarction. **(A)** Volcano plot of the associated dataset; **(B)** Venn diagram illustrating the intersection of the selected genes from the relevant datasets.

Acute myocardial infarction-related datasets (GSE60993, GSE127853, GSE218474, and GSE249812) were retrieved from the GEO database (Figure 1A). Differentially expressed genes (DEGs) were identified using a threshold of *P* < 0.05 and |Log2FC| ≥ 1. The Jvenn tool was used to generate a Venn diagram, revealing five AMI-associated DEGs: JUP, DUSP5, GLRX, VDAC3, and INSIG1 (Figure 2B).

### 3.2 Correlation analysis between DEGs and myocardial infarction biomarkers

**Figure 3:**
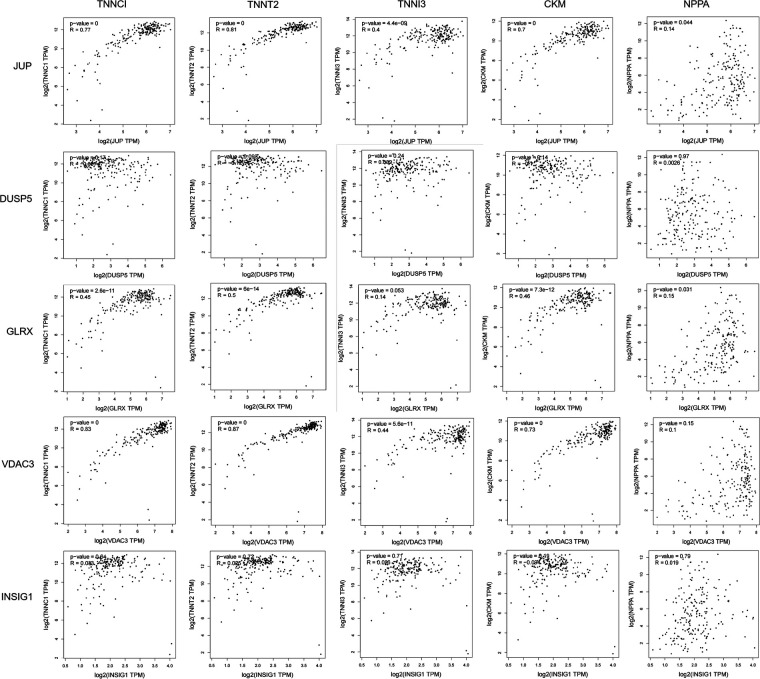
Correlation analysis between differentially expressed genes (DEGs) and myocardial infarction biomarkers, including Troponin, CK-MB, and BNP. Each scatter plot displays the relationship between a specific DEG (x-axis: gene expression, log2 TPM) and a biomarker (y-axis). The correlation coefficient (r) and *P*-value are noted in each subplot.

The troponin genes (TNNC1, TNNT2, TNNI3), the creatine kinase gene (CKM), and the brain natriuretic peptide gene (NPPA) are recognized biomarkers of myocardial infarction. Correlation analysis between the DEGs and these biomarkers was conducted using the GEPIA database (http://gepia.cancer-pku.cn/index.html). Notably, JUP and VDAC3 exhibited a high correlation with myocardial infarction biomarkers; however, JUP also demonstrated a significant correlation with TNNC1, TNNT2, TNNI, CKM, and NPPA (*P* < 0.05). Therefore, we selected JUP for further investigation, as illustrated in Figure 3.

### 3.3 Expression of the JUP gene in myocardial tissue

**Figure 4:**
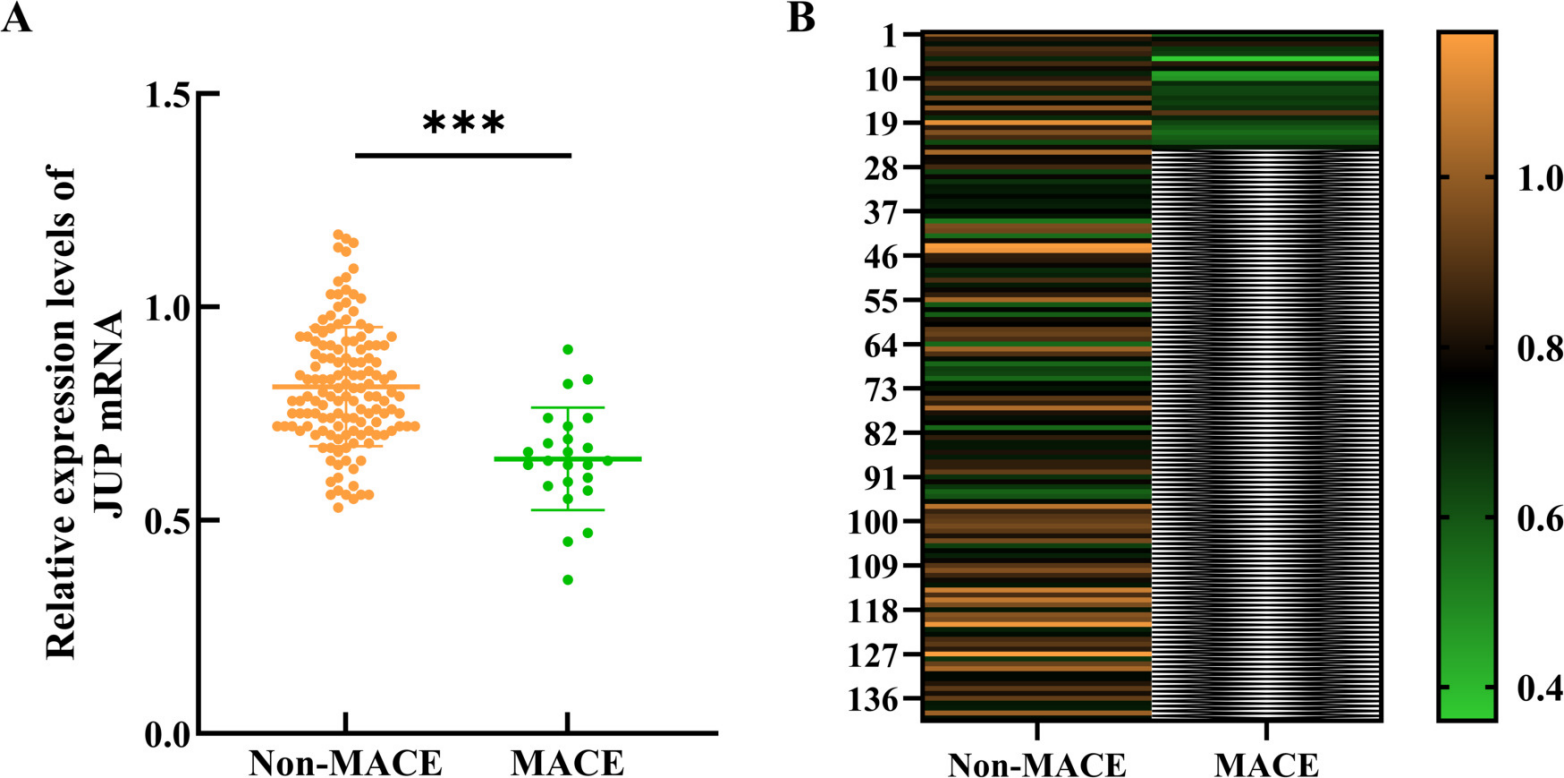
Expression of the JUP gene in myocardial tissue. **(A)** Scatter plot of myocardial JUP gene expression levels between the two groups; **(B)** Heatmap of JUP gene expression levels in myocardial tissue between the two groups. *** indicates *P* < 0.001 compared to the Non-MACE group. The color scale on the right indicates expression levels, with orange representing upregulated genes, green representing downregulated genes, and black denoting no significant change. The intensity of the colors corresponds to the magnitude of gene expression changes, where darker shades signify greater fold changes.

RNA was extracted from myocardial tissue and reverse transcribed into cDNA for qRT-PCR analysis of JUP gene mRNA expression. The study followed 24 patients over a period of 12 months, during which 24 major adverse cardiovascular events (MACE) occurred, resulting in an incidence rate of 14.6%. Comparison of JUP expression levels in myocardial tissue between the event and non-event groups revealed that the JUP expression level in the event group was 0.64 ± 0.12, significantly lower than that in the non-event group, which was 0.81 ± 0.14 (*t* = 5.603, *P* < 0.001), as shown in Figure 4.

### 3.4 Relationship between clinical pathological characteristics of acute myocardial infarction patients and adverse prognosis

A comparative analysis of the clinical data between the two groups of patients revealed no statistically significant differences in age, sex, BMI, history of hypertension, heart rate, atrial fibrillation, smoking history, history of diabetes, and LDL-C levels (*P* > 0.05). In contrast, when compared to the non-event group, the event group exhibited significantly longer door-to-balloon times, elevated levels of CK and TnI, higher postoperative TIMI flow grades, a greater proportion of patients with multiple culprit vessels, and lower expression levels of myocardial JUP, with statistical significance (*P* < 0.05), as presented in Table 2.

**Table 2:** Relationship between clinical pathological characteristics of acute myocardial infarction patients and adverse prognosis.

Clinical data		MACE (*n* = 24)	Non-MACE (*n* = 140)	$\chi^{2}/t$ 值	*P*-value
Age (years)		62.58 ± 8.50	62.67 ± 10.80	0.038	0.970
Gender (*n*/%)	Female	11/45.83%	64/45.71%	0.000	0.991
	Male	13/54.17%	76/54.29%		
BMI		25.95 ± 3.99	25.57 ± 4.16	0.435	0.666
Door-to-Balloon Time (h)		5.80 ± 1.69	4.06 ± 1.38	5.514	<0.001
Hypertension (*n*/%)	Yes	17/68.00%	109/77.86%	1.142	0.285
	No	8/32.00%	31/22.14%		
Heart Rate (beats/min)		80.75 ± 5.82	79.17 ± 5.01	1.393	0.166
Atrial Fibrillation (*n*/%)	Yes	8/33.33%	54/38.57%	0.239	0.625
	No	16/66.67%	86/61.43%		
Smoking History (*n*/%)	Yes	11/45.83%	63/45.00%	0.006	0.094
	No	13/54.17%	77/55.00%		
Diabetes (*n*/%)	Yes	14/58.33%	77/55.00%	0.092	0.761
	No	10/41.67%	63/45.00%		
EF (%)		48.00 ± 9.28	56.26 ± 8.70	4.068	<0.001
LDL-C (mmol/L)		3.44 ± 0.20	3.62 ± 0.56	1.512	0.132
CK (IU/L)		347.70 ± 69.49	252.17 ± 60.42	6.998	<0.001
TnI (pg/ml)		38.89 ± 5.98	28.90 ± 8.41	5.576	<0.001
TIMI ≤ Ⅱ grade	Yes	7/29.17%	76/54.29%	5.172	0.023
	No	17/70.83%	64/45.71%		
Multivessel Disease (*n*/%)	Yes	16/66.67%	50/35.71%	8.162	0.004
	No	8/33.33%	90/64.29%		

### 3.5 Multivariate Cox proportional hazards model of risk factors for postoperative adverse events in patients with acute myocardial infarction

Due to the clarity of the clinical data and the small sample size, we used the median to classify the continuous variable indicators with statistical differences in the univariate analysis. After assigning values to the clinically significant variables—door-to-balloon time, CK levels, TnI levels, postoperative TIMI flow grades, multivessel disease, and myocardial JUP levels—multivariate Cox Proportional Hazards model was conducted. The model exhibited a good fit, with the chi-square test indicating statistical significance (*P* < 0.05) (HR = 7.892; 95% CI = 1.818–34.269; *P* = 0.006). The results demonstrated that TnI levels, multivessel disease, and myocardial JUP levels are independent predictors of postoperative adverse events in patients undergoing PCI for acute myocardial infarction, as detailed in Tables 3, 4.

**Table 3:** Model variable assignment.

Study variables	Variable code	Assignment
MACE	Y	“1” = “Yes”, “2” = “No”
Door-to-Balloon Time (h)	X1	“1” = “≤4.3”, “2” = “>4.3”
EF (%)	X2	“1” = “<50”, “2” = “≥50”
CK (IU/L)	X3	“1” = “<262.84”, “2” = “≥262.84”
TnI (pg/ml)	X4	“1” = “<30”, “2” = “≥30”
TIMI ≤ Ⅱ grade	X5	“1” = “≤Ⅱ grade”, “2” = “>Ⅱ grade”
Multivessel disease (*n*/%)	X6	“1” = “Yes”, “2” = “No”
JUP	X7	“1” = “≤0.78”,“2” = “>0.78”

**Table 4:** Multivariate Cox proportional hazards regression analysis of risk factors for postoperative adverse events in patients with acute myocardial infarction.

Included Variables	β	SE	Wald $x^{2}$ value	*P*-value	HR (95% CI)
Door-to-Balloon Time (h)	0.877	0.532	2.715	0.099	2.403 (0.847–6.820)
EF (%)	−0.477	0.450	1.121	0.290	0.621 (0.257–1.501)
CK (IU/L)	0.907	0.610	2.212	0.137	2.477 (0.750–8.186)
TnI (pg/ml)	2.527	1.042	5.876	0.015	12.512 (1.622–96.507)
TIMI ≤ Ⅱ grade	0.860	0.551	2.434	0.119	2.363 (0.802–6.961)
Multivessel disease (*n*/%)	−1.204	0.522	5.330	0.021	0.300 (0.108–0.834)
JUP	−1.451	0.655	4.907	0.027	0.234 (0.065–0.846)

### 3.6 K-M method: risk factors for postoperative MACE in patients with acute myocardial infarction

**Figure 5:**
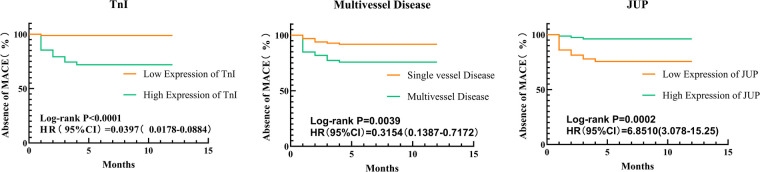
Kaplan-Meier survival analysis of postoperative major adverse cardiovascular events (MACE) in patients with acute myocardial infarction (AMI), stratified by (TnI, multivessel disease, JUP). The survival probability is plotted over time for high-risk (orange) and low-risk (green) groups. The log-rank test was used to assess statistical differences between groups.

Kaplan-Meier survival curve analysis revealed statistically significant differences between two groups, with Log-rank test results showing TnI (*x*^2^ = 23.47, *P* < 0.0001), Multivessel Disease (*x*^2^ = 8.351, *P* = 0.0039), and JUP (*x*^2^ = 16.93, *P* < 0.0001). These findings indicate that the incidence of MACE was reduced in the low TnI expression group compared to the high TnI expression group, in the single-vessel disease group compared to the multivessel disease group, and in the high JUP expression group compared to the low JUP expression group, as illustrated in Figure 5.

### 3.7 ROC curve analysis was performed to evaluate the predictive value of various factors for adverse prognosis in patients with acute myocardial infarction

**Figure 6:**
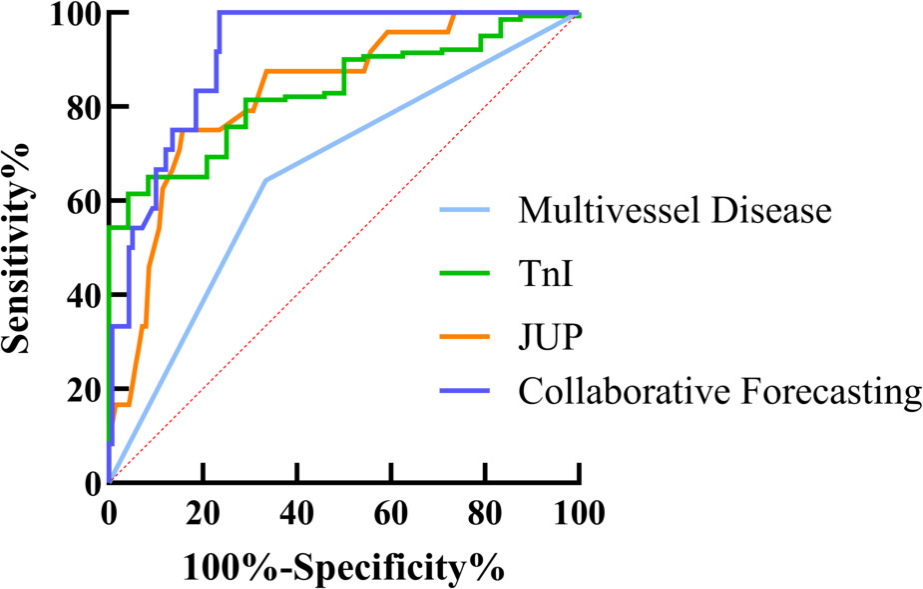
ROC curve analysis demonstrating the predictive value of (Multivessel disease, TnI, JUP and collaborative forecasting) for postoperative MACE in AMI patients. The area under the curve (AUC) for each factor is shown, with higher AUC values indicating better diagnostic performance. The diagonal red dashed line represents the reference line (AUC = 0.5, no predictive power).

Based on the results of the Multivariate Cox Proportional Hazards model, ROC curve analysis was performed for TnI, Multivessel Disease, and JUP. The findings indicated that all three factors can predict the risk of MACE following PCI in patients with acute myocardial infarction, with AUC values of 0.6548 for multivessel disease, 0.8316 for TnI, and 0.8286 for JUP. The sensitivity and specificity for each predictor were as follows: TnI—sensitivity 64.29%, specificity 66.67%; Multivessel Disease—sensitivity 40.71%, specificity 91.67%; and JUP—sensitivity 84.29%, specificity 75.00%. The combined predictive model yielded an AUC of 0.8785, with a sensitivity of 75.61% and a specificity of 96.00%, as presented in Table 5 and Figure 6.

**Table 5:** ROC curve analysis was performed to evaluate the predictive value of various factors for adverse prognosis in patients with acute myocardial infarction.

Factor	AUC	Std	95% CI	Sensitivity %	Specificity %	Youden's index
Multivessel Disease	0.6548	0.0605	0.5362–0.7733	64.29	66.67	0.3069
TnI	0.8315	0.0349	0.7631–0.9000	40.71	91.67	0.4596
JUP	0.8299	0.0435	0.7447–0.9152	75.00	84.29	0.5929
Collaborative Forecasting	0.9124	0.0234	0.8664–0.9583	100.00	76.43	0.7643

## 4 Discussion

In this study, we identified differentially expressed genes associated with acute myocardial infarction (AMI) through the GEO database, focusing on the expression of the junction protein JUP and its impact on postoperative prognosis. The findings indicated that JUP is significantly downregulated in myocardial tissue after PCI in AMI patients and is closely associated with adverse postoperative outcomes. The following sections will provide a detailed discussion of the main findings of this study, the functional role of JUP in cardiomyocytes, and its potential application as a clinical prognostic marker.

JUP is a crucial component of the adherens junction complex (16), primarily responsible for intercellular connectivity, particularly in the mechanical coupling and electrical signal transmission within cardiomyocytes (3). The structure of the adherens junction plays a vital role in maintaining the stability of cardiomyocyte membranes and the overall mechanical stability of myocardial tissue, especially during cardiac contraction and relaxation, ensuring tight physical connections between cells (17). Additionally, as an important adhesive molecule, JUP is key in maintaining cardiomyocyte morphology and facilitating cardiac electrical signal conduction (18). Studies indicate that the loss or dysfunction of JUP can lead to abnormal cell connections, potentially resulting in cardiomyopathies or functional impairments (19–22). Our research findings show that JUP expression is significantly downregulated in AMI patients compared to normal controls, suggesting its inhibition during the repair process following myocardial injury.

JUP, as an adhesion connexin, is crucial in maintaining the stability of cardiomyocyte connections and signal transduction. Studies have shown that downregulation of JUP may lead to disruption of cell connections, increased risk of arrhythmia, and may influence myocardial fibrosis and apoptosis through the Wnt/β-catenin signaling pathway. These mechanisms may explain the potential role of JUP in AMI prognosis. However, compared with other AMI-related biomarkers, JUP is still in the early research stage and its clinical value needs to be further validated. For example, high-sensitivity C-reactive protein (hs-CRP) mainly reflects systemic inflammatory states, while GDF-15 is involved in cellular stress responses, and JUP may be more focused on cell connection stability and myocardial fibrosis. Compared with soluble ST2 (sST2), JUP may have higher cell specificity, but there is no widely used clinical detection method.

Furthermore, this study found significant differences in door-to-balloon time, CK levels, TnI levels, postoperative TIMI flow grades, and the proportion of multi-vessel lesions between the two groups. A longer door-to-balloon time indicates prolonged myocardial ischemia, leading to poorer reperfusion outcomes and increased irreversible damage to cardiomyocytes, which in turn enlarges the area of myocardial necrosis and raises the probability of MACE in patients. The TIMI flow grade is an important tool for assessing coronary artery reperfusion; lower postoperative TIMI grades indicate poorer recovery of coronary blood flow and a longer duration of myocardial ischemia, which increases the risk of cardiomyocyte damage and the likelihood of adverse outcomes. CK and TnI are common biomarkers for myocardial infarction, with elevated levels correlating positively with the extent of myocardial damage; such patients are often more susceptible to experiencing MACE.

Furthermore, this study found significant differences in door-to-balloon time, CK levels, TnI levels, postoperative TIMI flow grades, and the proportion of multi-vessel lesions between the two groups. A longer door-to-balloon time indicates prolonged myocardial ischemia, leading to poorer reperfusion outcomes and increased irreversible damage to cardiomyocytes, which in turn enlarges the area of myocardial necrosis and raises the probability of MACE in patients. The TIMI flow grade is an important tool for assessing coronary artery reperfusion; lower postoperative TIMI grades indicate poorer recovery of coronary blood flow and a longer duration of myocardial ischemia, which increases the risk of cardiomyocyte damage and the likelihood of adverse outcomes. CK and TnI are common biomarkers for myocardial infarction, with elevated levels correlating positively with the extent of myocardial damage; such patients are often more susceptible to experiencing MACE.

Acute myocardial infarction (AMI) is a severe cardiovascular condition typically caused by acute obstruction of the coronary arteries, leading to myocardial ischemic necrosis (23). During ischemia, cardiomyocytes undergo complex biological changes, including membrane damage, apoptosis, and inflammatory responses (24–27). Our analysis revealed a significant decrease in JUP expression in the myocardial tissue of AMI patients, potentially linked to the disruption of intercellular junction structures (28). Additionally, research by Panigrahy et al. (29) demonstrated that ACM disease features can be reversed in rat ventricular myocytes expressing mutant JUP by the pro-resolving epoxy fatty acid (EpFA) 14,15-eicosatrienoic acid (14-15-EET), suggesting that JUP may influence cardiomyocyte survival and death in the context of AMI by modulating cell adhesion molecules, inflammatory pathways (20), and apoptosis mechanisms.

This study utilized Kaplan-Meier survival analysis and Cox proportional hazards regression to identify JUP, TnI, and Multivessel Disease as independent risk factors for adverse prognosis following PCI in AMI patients. TnI, a traditional marker of myocardial injury, has established theoretical foundations in prior research. Multivessel disease often indicates more extensive myocardial hypoperfusion and ischemic injury, frequently associated with complex atherosclerotic changes that may impact critical branches of the coronary arteries (30), complicating postoperative vascular remodeling and healing processes (31). Based on the analysis of JUP expression and its relationship with prognosis in AMI patients, we propose that JUP could serve as a potential biomarker for assessing prognosis in AMI. Previous studies have demonstrated the importance of the integrity of the cardiomyocyte adherens junction complex for cardiac function, and JUP, as a key component (32), may reflect the severity of myocardial damage (33). Our findings confirm the correlation between JUP expression levels and postoperative outcomes in AMI, such as heart failure, recurrent myocardial infarction, and arrhythmias, consistent with the results of Li et al. (34) and Marian AJ (35). This suggests that JUP has advantages in assessing the long-term prognosis of AMI patients, as it can directly reflect changes in cardiomyocyte connectivity and structure. Monitoring JUP levels in AMI patients may aid in the early identification of high-risk individuals, thereby informing personalized treatment strategies.

The results of this study indicate that the AUC for multivessel disease is 0.6548, reflecting a weak predictive ability with limited sensitivity and specificity. In contrast, TnI demonstrated an AUC of 0.8316, showing high specificity (91.67%) that aids in effectively ruling out adverse events, although its relatively low sensitivity (40.71%) may lead to missed diagnoses. JUP exhibited a similar predictive capacity to TnI, providing additional support. Most importantly, the combined analysis revealed an AUC of 0.8785 for the three factors, with specificity significantly elevated to 96.00%. This suggests that the integrated use of these markers can more effectively identify high-risk patients, thus informing personalized treatment strategies.

The clinical feasibility of JUP as a potential biomarker for prognostic prediction of AMI needs further evaluation. Currently, JUP can be detected by qPCR, ELISA, and immunohistochemistry (IHC), but its sensitivity, specificity, and cost-effectiveness need to be optimized. In addition, clinical promotion faces standardization challenges, such as the inconsistency of different laboratory testing methods, and the accuracy of reference ranges. In addition, in terms of AMI risk assessment, GRACE score and TIMI score are widely used in clinical practice, and subsequent studies should be combined with JUP to predict AMI, so as to improve the diagnostic efficiency of AMI.

Despite revealing the potential role of JUP in the prognosis of AMI through multi-omics data analysis and clinical validation, this study has several limitations. First, the sample size is relatively small; while our findings are statistically significant, it is essential to validate these conclusions in larger cohorts. Besides, univariate and multivariate Cox regression analyses were used to assess the prognostic value of JUP. Although some confounders (such as age, hypertension, diabetes, etc.) have been adjusted, residual confounders (such as unmeasured inflammatory factors) may still exist. Future multicenter studies with larger cohorts will prioritize subgroup analyses stratified by infarction type (STEMI/NSTEMI), diabetic status, and revascularization patterns to refine JUP's clinical utility. Second, this research primarily relies on data from the GEO database, necessitating further validation through independent clinical samples in the future. Additionally, the specific molecular mechanisms of JUP in AMI require further investigation, particularly regarding its interactions with other signaling pathways.

Future studies should focus on the role of JUP in the myocardial repair process following infarction, especially its regulatory processes in cardiomyocyte adhesion and the Wnt/β-catenin signaling pathway. Furthermore, integrating multi-omics technologies, such as proteomics and metabolomics, to explore the interactions between JUP and other molecules may help elucidate its comprehensive role in AMI. As research progresses, JUP holds promise as an important molecular target for prognostic assessment and personalized treatment in AMI, providing new insights for the clinical management of these patients.

## 5 Conclusion

In summary, this study reveals the significant role of JUP in AMI through bioinformatics analysis and its potential as a prognostic biomarker. The downregulation of JUP expression is closely associated with adverse outcomes in AMI patients, suggesting it could serve as an important biomarker for prognosis assessment. However, the clinical application of JUP faces challenges related to technical translation, mechanistic research, and clinical validation. Future studies should focus on exploring the mechanisms of JUP and developing simpler, faster detection methods to facilitate its broader use in clinical settings.

## Data Availability

The original contributions presented in the study are included in the article/Supplementary Material, further inquiries can be directed to the corresponding author.
